# Effectiveness of targeted post-acute interventions and follow-up services for sepsis survivors: a systematic review

**DOI:** 10.1186/s13054-025-05585-3

**Published:** 2025-08-08

**Authors:** Barbora Bircak-Kuchtova, Norman Rose, Christian Geis, Kathrin Finke, Mathias W. Pletz, Ha-Yeun Chung, Carolin Fleischmann-Struzek

**Affiliations:** 1 Section Translational Neuroimmunology, Department of Neurology, Jena University Hospital, Am Klinikum, 07747 Jena, Germany; 2 Institute of Infectious Diseases and Infection Control, Jena University Hospital/Friedrich-Schiller-University Jena, Jena, Germany; 3 Center for Sepsis Control and Care, Jena University Hospital, 07747 Jena, Germany; 4 Department of Neurology, Jena University Hospital, 07747 Jena, Germany

**Keywords:** Sepsis, Post-sepsis syndrome, Post-acute care, Sepsis sequelae, Long-term outcomes of sepsis

## Abstract

**Background:**

The majority of sepsis survivors suffer from significant long-term consequences, including cognitive, psychological, and physical impairments. Despite growing recognition of these challenges, there is a lack of robust evidence regarding effective post-acute interventions to improve long-term outcomes. This systematic review aims to compile the present evidence on the effectiveness of post-acute interventions and follow-up services on patient-relevant long-term outcomes of sepsis survivors.

**Methods:**

PubMed, Web of Science and ClinicalTrials.gov were searched for relevant publications from 01/2013 until 08/2024. Studies evaluating the effect of targeted post-acute interventions and follow-up services compared to usual care were included. Risk of bias was assessed using the RoB2- and ROBINS-I tool.

**Results:**

Fourteen studies including 383,680 patients from high-income-countries were identified. All included studies showed either a moderate risk of bias (non-randomized studies) or some concerns (randomized trials), primarily due to residual confounding, suboptimal blinding and outcome assessment. Interventions varied substantially in terms of measures, implementation time and outcomes addressed. Rehabilitation interventions were associated with long-term survival benefits until 10 years after sepsis according to three observational studies. Additionally, one randomized controlled trial with minimization found that an 8-week exercise-based intervention improved the anaerobic threshold in sepsis survivors. Interventions (*n* = 7) targeting care coordination and follow-up bundles led to reductions in rehospitalization rates and mortality until 12 months post-discharge and were associated with improvements in long-term physical function and PTSD symptoms. An ICU-specific virtual reality-based intervention may reduce symptoms of PTSD and depression up to six months after exposure.

**Conclusion:**

Post-acute interventions, such as care coordination, bundle approaches, and rehabilitation can improve patient-relevant outcomes in sepsis survivors. However, the overall number of existing studies is small, all studies may be affected by certain forms of bias and for some domains of post-sepsis impairment no specific interventions have yet been identified. Therefore, further high-quality prospective follow-up studies are needed to strengthen the evidence regarding the effectiveness and acceptability of interventions across all domains of post-sepsis impairments, particularly cognitive impairments.

**Supplementary Information:**

The online version contains supplementary material available at 10.1186/s13054-025-05585-3.

## Introduction

Sepsis is a life-threatening condition due to a dysregulated immune response, leading to widespread inflammation, tissue damage, and organ dysfunction. It represents a major global health challenge and is associated with an estimated 20% of global deaths [1]. In survivors, sepsis often results in long-term impairments, which can lead to functional dependency, reduced quality of life, and frequent rehospitalizations due to cardiovascular complications or new or recurrent infections [2, 3]. Therefore, these long-term consequences impose a significant burden not only for relatives and caregivers, but also on the healthcare system as a whole [4].

Long-term impairments, described in recent literature as post-sepsis syndrome (PSS), encompass a broad range of physical, cognitive and psychological symptoms. These may include chronic fatigue, muscle weakness, sleep disturbances, deficits in memory and attention, as well as anxiety and depressive symptoms. Even three years after the acute illness, 91.2% of survivors were affected by at least one new physical post-sepsis impairment, and 57.9% and 40.9% had new cognitive and psychological symptoms, respectively [5]. While PSS may affect both individuals who underwent intensive care treatment and those who did not, it is more commonly observed in patients who have experienced critical illness and intensive care treatment [6].

Given the significant burden of long-term sequelae, interdisciplinary follow-up care is of utmost importance. Therefore, the 2021 Surviving Sepsis Campaign guidelines included new recommendations for sepsis follow-up care. Besides the recommendation for screening for long-term impairments and providing information about sepsis and its sequelae, the guidelines highlight the importance of follow-up care to address physical and cognitive impairments, as well as emotional distress, after hospital discharge [7]. However, an International Sepsis Forum colloquium highlighted the scarcity of effective symptom-guided interventions and aftercare for the survivor population [8]. Thus, specific concepts for targeted aftercare for sepsis survivors are still limited [9] and not further specified in the Surviving Sepsis Guidelines. To provide insights into these concepts, we conducted a systematic synthesis of studies on the impact of post-sepsis follow-up interventions on survivor outcomes.

## Materials and methods

### Guidelines and protocol registration

The systematic review protocol was prospectively registered in the International Prospective Register of Systematic Reviews (PROSPERO; identification number CRD42024534497; available at https://www.crd.york.ac.uk/PROSPERO/view/CRD42024534497). Reporting follows the PRISMA (Preferred Reporting Items for Systematic Reviews and Meta-Analyses) guidelines for systematic reviews and meta-analyses [10].

### Eligibility criteria

We included studies evaluating the effects of targeted post-acute interventions and follow-up services for adult sepsis survivors, where outcomes were compared to those receiving usual care. Studies were included if they reported the proportion of sepsis patients and at least approximately 50% of the study population had a confirmed sepsis diagnosis. Observational and interventional studies were included. We excluded reviews and case reports, as well as studies written in other languages than English and German.

### Search strategy

PubMed, Web of Science and ClinicalTrials.gov databases were searched for studies published between January 1 st 2013 and August 30th 2024 using a pre-planned, systematic, reproducible search strategy. Search terms incorporated keywords for ‘sepsis’, ‘post-intensive care syndrome’ or ‘post-sepsis syndrome’, and ‘intervention’, ‘rehabilitation’ or ‘follow-up’. The full search strategy is available in the Supplement. Moreover, reference lists of the retrieved articles fulfilling the inclusion criteria were searched for additional studies.

### Study selection and data extraction

Eligibility assessment was performed in a standardized manner. Two independent reviewers screened titles and abstracts and consecutively full-texts using Covidence, a web-based tool for conducting systematic reviews (Covidence systematic review software, Veritas Health Innovation, Melbourne, Australia. Available at www.covidence.org*).* Conflicts were resolved by involving a third reviewer for arbitration.

The following data were extracted: author details, year of publication, year of study, country, study design, study setting, content and duration of intervention, number of participants (intervention and control group), length of post-discharge follow-up, time of outcome measurement, outcome and results. Firstly, the data was extracted by one reviewer and revised by at least one other reviewer. As previously, disagreements were resolved by consensus.

### Assessment of risk of bias

For the randomized controlled trials (RCT), the Cochrane RoB 2 tool was used to assess the risk of bias (RoB) [11], with ratings of ‘low risk‘, ‘some concerns‘ or ‘high risk‘ of bias. The risk of bias in non-randomized studies was assessed using ROBINS-I-Tool, with judgments in each domain categorized as ‘very low‘, ‘low‘, ‘moderate‘ or ‘high‘ [12].

### Assessment of intervention reporting and fidelity

Intervention reporting and fidelity was systematically assessed using the Template for Intervention Description and Replication (TIDieR) checklist [13].

### Data synthesis

Due to the heterogeneity in interventions and outcomes measured, no meta-analysis could be performed. The results of randomized and non-randomized studies are therefore reported descriptively in the ‘Summary of findings’ table. The included studies report different effect measures due to different outcomes with different scales (binary and metric outcomes) and different statistical approaches used for data analysis. Unadjusted and adjusted conditional odds ratio were most frequently reported for binary outcomes. An adjusted risk reduction was reported by one study [14]. Unadjusted and adjusted hazard ratios were reported when time-to-event analyses were used [15, 16]. Both odds ratios and hazard ratios suffer from the problem of non-collapsibility, which means that (a) unconditional and conditional odds ratios are not numerically comparable and (b) conditional odds ratios are also not comparable among each other if the set of covariates differ across models/studies [17]. Therefore, no common metric across studies and models could be established for the different effect measures of binary outcomes. Standardized mean differences (SMD) for metric outcomes such as Cohen’s d have been rarely provided in the reviewed studies. Therefore, we derived or approximated SMDs from the numerical data and quantities reported in the original studies. Ideally, Cohen’s d could be computed as the mean difference MD(*Y*) of an outcome *Y* between control and treatment group divided by the within-group standard deviation SD_*within*_(Y). Such computation was possible for the study of Mohammad et al. [18] with the pooled standard deviation as an estimate of SD_*within*_(Y). However, SD_*within*_(Y) could often not be derived from the publications. Therefore, different alternative quantities were used for standardization of the raw effect. For the study of Batterham et al. [19], the pooled standard deviation SD_*pooled*_(Y_pre_) at the baseline in a pre-post design was used. The studies by Gawlytta et al. [20] and Schmidt et al. [21, 22] also used a pre-post design with the change score CS = Y_post_– Y_pre_ as the outcomes in the statistical analyses. Accordingly, the effect measure is the mean difference MD(CS) in the change scores between treatment and control group. The SMD (e.g., Cohen’s d) of the change score was reported by Gawlytta et al. [20] only. Schmidt et al. [21, 22] merely presented the unstandardized difference *M*_treatment_(CS) - *M*_control_(CS) in the mean change scores of the treatment and control group. We calculated the pooled standard SD_*pooled*_(Y_pre_) from the paper for computing the SMD. However, Schmidt et al. [21, 22] reported means and mean differences of the secondary outcomes (a) activities of daily living (ADL), (b) physical function (XSMFA-F), and (c) disability (XSMFA-B) 6 months and 24 months after treatment in an intensive care unit (ICU). Hence, Cohen’s d of these outcomes at the two time points instead of change scores could be calculated. The required pooled standard deviations were derived either from the standard errors or 95% confidence intervals (CI) of the group means or the interquartile ranges (IQR) of the outcomes in both groups under the assumption of normality.

## Results

### Study description

**Fig. 1:**
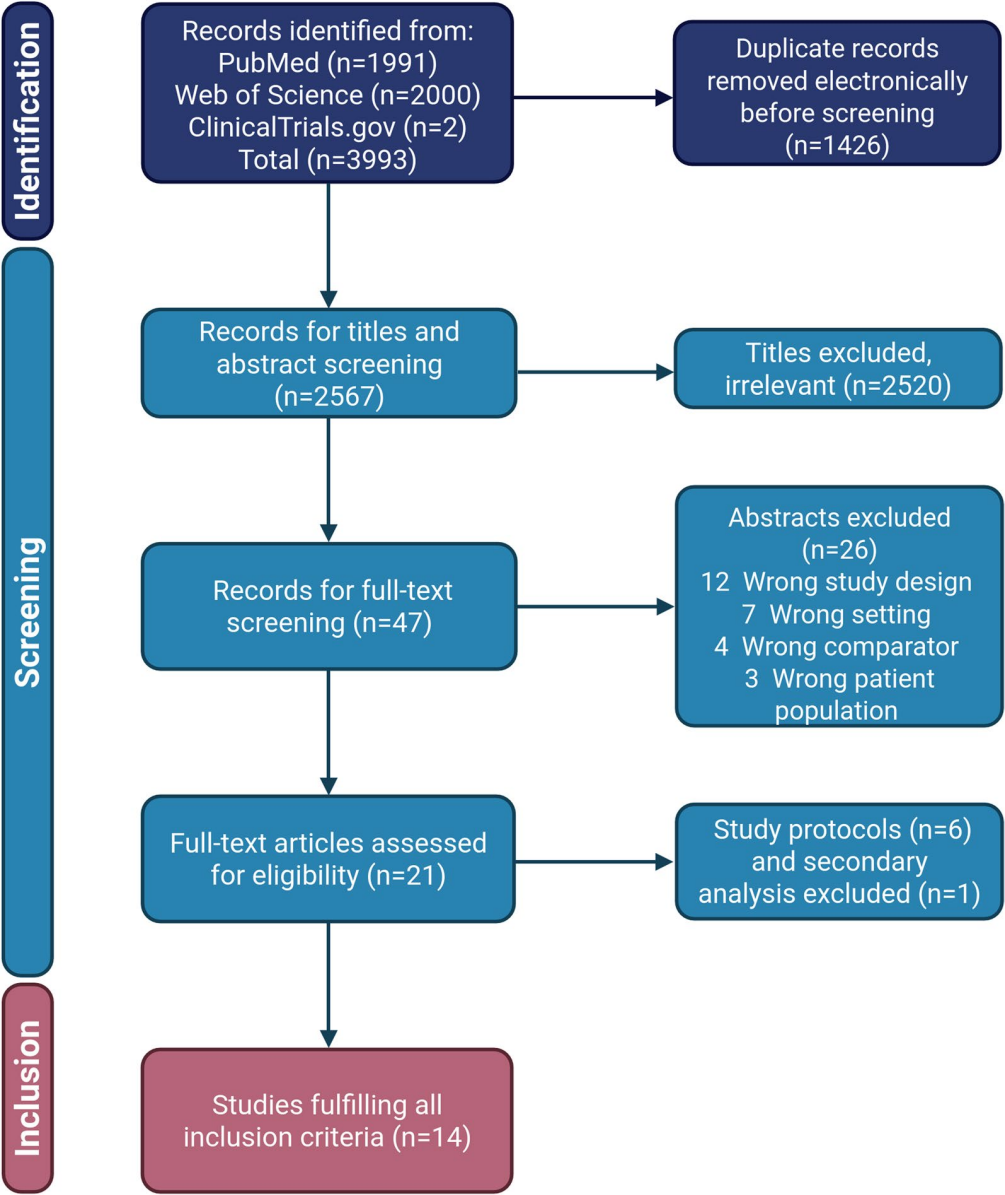
Flow of study inclusion (created in https://BioRender.com)

Of 2567 unique records identified by our search strategy, 21 studies met our inclusion criteria (Fig. 1). Out of these, six were study protocols and one was a secondary analysis of an included study. Consequently, 14 studies were included in the systematic review.

The characteristics of included studies are presented in Table 1, for characteristics of study protocols see eTable 1 in the Supplement. Studies originated from five countries, all of which were high-income-countries. Eight studies were controlled trials, while six studies were observational cohort studies.

#### Patient population and sepsis definition

In summary, studies included 383,680 patients, with a range between 25 and 167,948 patients in the individual studies. Across the included studies, six focused on ICU-treated sepsis patients, five on hospital-treated patients, and three recruited patients treated in the emergency department (ED). Sepsis was diagnosed according to differing criteria among the studies: criteria of the American College of Chest Physicians/Society of Critical Care Medicine (ACCP/SCCM) for severe sepsis [21–24]; explicit ICD-codes or implicit ICD-code combinations [14–16, 25, 26]; combined criteria of diagnosed infection and hypotension requiring the administration of a vasopressor or a blood lactate level ≥ 3.0 mEq/L [27]; self-reported sepsis [20]; or criteria of suspected sepsis by as antibiotic therapy or bacterial culture order within 24 h of ED arrival, combined with a readmission risk probability ≥ to 20% or mortality risk probability ≥ 10% [28–30]. Two studies did not report specifically the criteria used to define sepsis [18, 19].

#### Setting and time of intervention

**Fig. 2:**
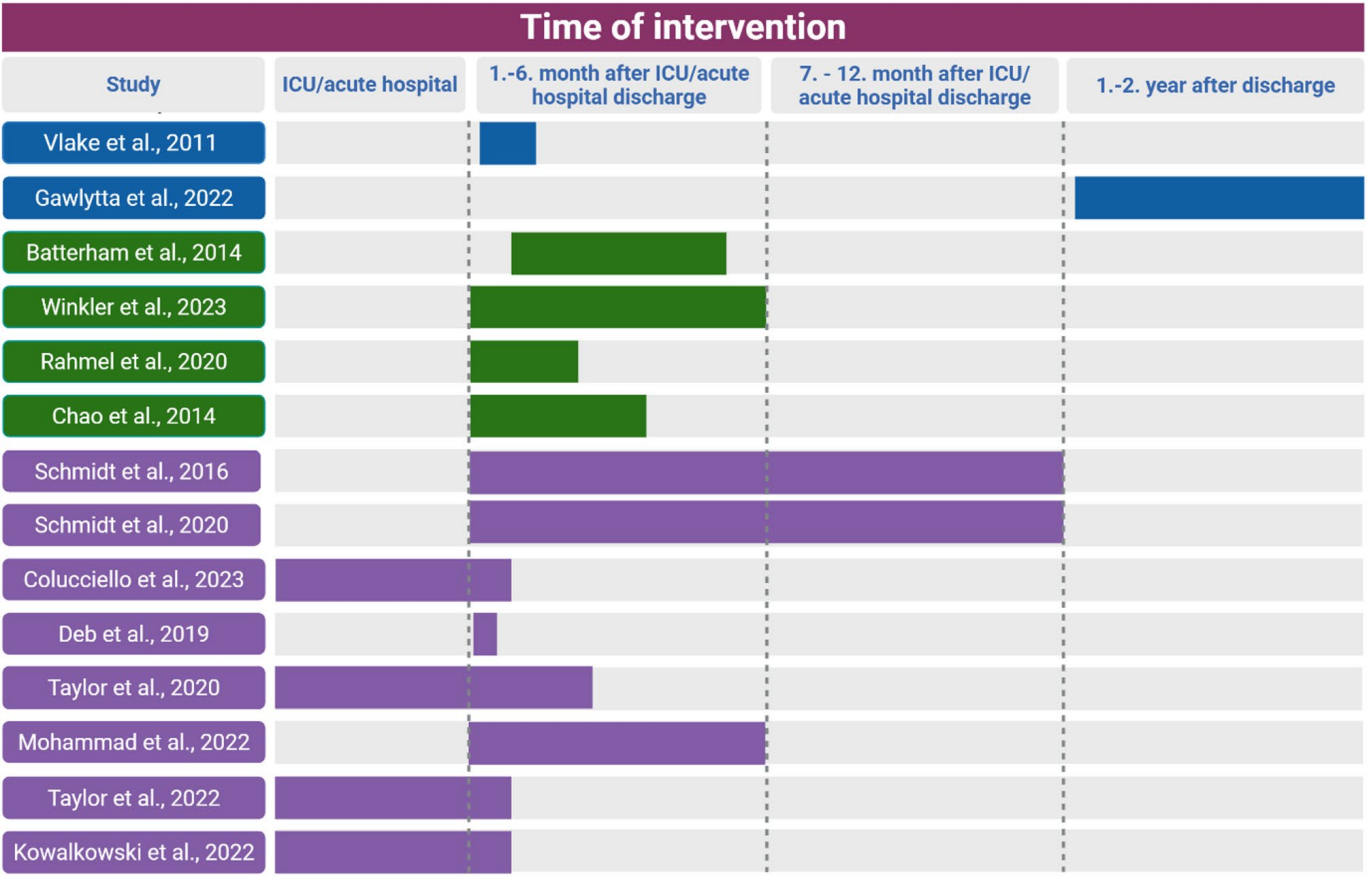
Implementation Period of the Interventions. Interventions focused on mental health are marked in blue; those including rehabilitation and physical exercise are marked in green; and studies using post-sepsis care coordination or bundle approaches as well as pharmacist-led interventions are marked in violet. (created in https://BioRender.com)

Studies targeted cognitive impairments (*n* = 2), mental health impairments (*n* = 4), physical impairments (*n* = 3), readmissions, level of nursing care, mortality, total health care costs, and health-related quality of life as primary outcomes (Table 1). Most studies were conducted in clinics, remotely or at home. Time of implementation of follow-up interventions in the course of aftercare ranged between directly after discharge from the intensive care unit and 1.8 years post discharge (median), as presented in Fig. 2.

### Risk of bias

All non-randomized studies [14–16, 18, 25, 26] were judged to have a moderate risk of bias, primarily due to concerns related to residual confounding and participant selection (Table 2).

Among the randomized controlled trials, all studies [19–22, 27–29, 31] were rated as having some concerns regarding risk of bias (Table 3). These concerns mostly related to deviations from intended interventions and potential biases in outcome measurement and reporting inherent to subjective outcomes such as quality of life or mental health. In addition, the absence of blinding introduced a risk of performance and detection bias. Finally, attrition due to loss to follow-up or death was common. However, none of the studies were assessed as having a high risk of bias.

In addition to the bias domains assessed by RoB 2 and ROBINS-I, selection bias towards less severely affected patients was found in most of the included studies. Participation in follow-up intervention often required a minimum level of physical and cognitive functioning, leading to systematic exclusion of more impaired patients, e.g. with nursing care dependency (e.g [14, 25])., cognitive impairments (e.g [15, 21, 27])., or psychological impairments/treatment (e.g [20, 27]).

### Assessment of intervention fidelity of RCTs

The TIDieR checklist is provided in e Table 2 in the Supplement for all RCTs. While most studies provided basic information on the intervention rationale, procedures, and mode of delivery, several key elements were frequently underreported. In particular, details regarding the training and expertise of those delivering the intervention, strategies to ensure fidelity and adherence, tailoring or adaptations over time, and whether the intervention was delivered as planned were often either missing or inadequately described.

### Effectiveness of post-acute interventions and follow-up services

Table 4  summarizes the findings of the included studies. The studies are described below according to their primary domain of focus, i.e. mental health interventions (*n* = 2), rehabilitation and physical exercise interventions (*n* = 4) and studies focusing on care coordination, bundle approaches (*n* = 7) or pharmacist-led interventions (*n* = 1).

#### Interventions with focus on mental health

Controlled trials: Two randomized trials evaluated the effects of post-discharge intervention on the mental health of sepsis survivors using technology-delivered psychological interventions, examining a total of 67 patients. A study from the Netherlands investigated the use of virtual reality (VR) to simulate ICU experience for sepsis survivors [27]. The ICU-VR content was designed specifically to address patients’ memory gaps and offer context for distressing memories. The main focus of ICU-VR was to show and explain the ICU environment, inform about devices, noises, necessity of intravenous catheters, mechanical ventilation and other invasive procedures as well as to introduce the treatment team and ICU workflow. Patients were also provided with information about sepsis [27]. The intervention was initiated a median of 10 days after ICU discharge and all included patients completed at least one VR-session with frequency varying among participants. At six months, a lower proportion of patients in the ICU-VR group suffered from post-traumatic stress disorder (PTSD) compared to those in the control group, who used a non-specific VR (measured by IES-R, 8.7% vs. 47.4%, *p* = 0.01). The second study focused on mental impairment was an intervention study involving spouses of patients in internet-based therapist-led cognitive behavioral writing therapy (iCBT). At the time of inclusion, patients were a median of 1.8 years post-ICU treatment. The study found no evidence of the efficacy of iCBT in reducing PTSD symptom severity when compared to a waiting-list control group [20]. ICBT appeared to be safe and applicable, but drop-out rates were high (9/50 randomized).

Observational studies: No observational study with this focus was included.

#### Interventions with focus on rehabilitation- therapy and physical exercise

Controlled trials: In a randomized controlled trial using minimization to ensure group balance, which examined the effect of a hospital-based physiotherapist-supervised aerobic training among older adults with ICU-treatment due to sepsis or trauma, a beneficial effect on the anaerobic threshold in the intervention group was found (SMD = 0.58 (95% CI = 0.13 to 1.03)). The 8-week intervention consisted of two weekly, physiotherapist-led hospital sessions (40 minutes each) on a cycle ergometer at moderate intensity, with progressively increasing resistance. Additionally, one unsupervised session of the same duration and intensity was recommended per week [19]. Around 50% of participants completed all 16 supervised and 8 unsupervised sessions; with a mean of 12 and 6 sessions per patient, respectively.

Observational studies: In three observational studies examining rehabilitation and physical exercise (*n* = 3; 156,962 patients included), which identified sepsis using ICD-codes, mortality was the most examined outcome. A German retrospective matched cohort study found a significantly higher long-term survival rate (specifically 13–36 months after sepsis) in adult patients who underwent rehabilitation treatment in an inpatient rehabilitation clinic (adjusted OR 1.2 (CI = 1.1 to 1.3)) compared to those who did not [25]. Treatment duration and therapies could vary between patients and could not be further specified. Another German study also confirmed a survival benefit up to 5 years post-sepsis in patients with inpatient rehabilitation, compared to those who did not receive any inpatient rehabilitation clinic treatment after discharge from the hospital (adjusted HR 0.81, 95% CI = 0.77 to 0.85). The content of rehabilitation therapies could vary between patients depending on the individual focus of the rehabilitation facility [15]. A Taiwanese retrospective matched cohort study observed a positive effect of rehabilitation treatment within 90 days on survival up to 10 years post-sepsis, with an inverse association of rehabilitation with 10-year mortality (adjusted HR, 0.94; 95% CI = 0.92 to 0.97) as well as an association of greater frequency of rehabilitation courses with a lower risk of mortality (≥ 3 vs. 1 course of rehabilitation: adjusted HR, 0.82). Their rehabilitation program included structured exercises targeting strength, mobility, daily activities, cardiovascular endurance, and functional capacity, along with occupational and communication therapy. A rehabilitation dose was defined as a visit to a physiatrist that initiated a physiotherapist-led course consisting of six sessions (30–60 min each) over one month. Session frequency was determined based on the patient’s medical history, clinical condition, and tolerance. Additionally, patients were encouraged to engage in home-based exercises independently and/or with the support of family members on days when they did not receive rehabilitation guided by a physical therapist [16].

#### Results of studies using post-sepsis care coordination, bundle approaches

In total, seven studies evaluated the effect of care coordination and bundle approaches on sepsis long-term outcome including 171,550 patients.

RCTs: Five studies had a RCT design, of which three were analyses of the STAR (Sepsis Transition and Recovery) intervention and two were analyses of the SMOOTH (Sepsis survivors monitoring and coordination in outpatient health care) study. The STAR intervention included surveillance and treatment of new health problems as well as chronic conditions, medication review and adjustment together with a focus on care coordination, including palliative care when appropriate in patients with suspected sepsis in the ED. The intervention delivery was led by a registered nurse specialized in supporting sepsis survivors. The navigator’s primary responsibilities included facilitating care planning and self-management, ensuring proactive follow-up, and enhancing engagement among patients, healthcare providers, and the community. Results showed a beneficial effect on 30-day mortality as well as on readmission in the intervention group (death or readmission 30 days post-discharge: adjusted OR, 0.80; 95% Cl = 0.64 to 0.98) [28]. Furthermore, after 12 months, the intervention group had a lower combined mortality or readmission rate with an adjusted OR of 0.70 (95% CI = 0.50 to 0.98) [31]. The beneficial effect of the intervention was also observable in the subgroup of patients discharged to post-acute care [29]. A German multicenter RCT with 24-month-follow-up among ICU-treated sepsis patients according to the sepsis-1 definition (former severe sepsis) focused on proactive patient symptom monitoring and training, as well as clinical decision-making and training of primary care physicians in evidence-based post-sepsis-care [21, 22]. The study did not show improvements in mental-health related quality of life (primary outcome), but a better physical functioning and improvements in ADLs in the intervention group after six months (XSFMA-F (Extra Short Musculoskeletal Function Assessment regarding physical function), SMD: −0.25 (95% CI = −0.48 to −0.02), XSFMA-B (assessing disability), SMD − 0.27 (95% CI = −0.50 to −0.04), ADL impairments, SMD 0.27 (95% CI = 0.04 to 0.50)). Additionally, the 24-months follow-up analysis after discharge revealed an increase of PTSD symptoms in the control, however, not in the intervention group, indicating a potential protective benefit of the intervention (Posttraumatic Symptom Scale (difference between baseline and 24-months follow-up values), SMD(CS) = 0.17 (95% CI = −0.14 to 0.49)) [21, 22].

Observational studies: Two retrospective cohort studies assessed the impact of care coordination and bundled interventions. A study from the United States (US) examined the effect of both an outpatient follow-up visit in the first week after hospital discharge and early home health visits in hospital-treated sepsis patients (defined by explicit or implicit ICD codes or code combinations) discharged to home health. The first nursing visit was required within 2 days of hospital discharge, and at least one follow-up visit during the first week post-discharge. The combination of nursing and physician visit was associated with lower readmission rate 30 days after discharge (7% risk reduction; 95% CI = 2 to 12) [14]. Furthermore, post-sepsis care elements during hospitalization and after discharge, including medication management, screening for deficits, monitoring preventable causes of health deterioration and aligning treatment with patients’ preferences, were associated with lower mortality and hospital readmission within 90 days in adults with diagnosed sepsis based on ICD codes. Specifically, the implementation of at least two of the aforementioned elements was associated with lower odds of 90-day mortality or hospital readmission compared to having none or only one element documented (two elements vs. none: OR 0.26; 95% CI = 0.10 to 0.69; three vs. none: OR 0.28; 95% CI = 0.11 to 0.72; four vs. none: OR 0.12; 95% CI = 0.03 to 0.50) [26].

#### Pharmacist-involvement in aftercare

Observational studies: In a retrospective cohort study, the presence of a pharmacist in an interprofessional team in an ICU-recovery clinic was evaluated and showed a significant decrease in medication-related problems in the intervention group including 104 survivors of ICU-treated sepsis or septic shock and/or respiratory failure at the 6-month follow-up visit compared to baseline, but no significant difference to the control group (number of medication-related problems: SMD 0.04 (95% CI = −0.27 to 0.35)) [18].

## Discussion

### Summary of main results

This systematic review included 14 studies evaluating the effectiveness of different post-sepsis interventions, including mental health support, physical rehabilitation, care coordination, and pharmacist-led programs. Evidence from randomized controlled trials and observational studies suggests that structured post-acute care– particularly rehabilitation and coordinated follow-up interventions– may improve long-term outcomes in sepsis survivors, specifically physical functions, mental health, and survival. While technology-delivered psychological interventions showed promise in reducing symptoms of PTSD and depression, the findings were mixed, and the evidence remains limited. Rehabilitation programs, especially those delivered in inpatient settings or with repeated sessions, were consistently associated with improved survival up to 10 years post-sepsis. Care coordination and bundled approaches demonstrated benefits in reducing mortality and hospital readmissions. Overall, most studies showed either moderate risk of bias or some concerns, indicating a need for further high-quality research to confirm the effectiveness and generalizability of these interventions.

### Completeness of evidence

The available evidence on the effectiveness of post-sepsis interventions is incomplete and limited in both scope and methodological quality. While a range of interventions has been investigated, the number of high-quality studies remains low. Several of the included randomized trials were pilot or feasibility studies with small sample sizes, limiting the precision of effect estimates and increasing the risk of small study effects. All included studies were conducted in high-income countries and any results are limited to settings with comparable healthcare resources. Across study designs, risk of bias was a concern: all randomized trials were judged as having ‘some concerns’ and all non-randomized studies as having ‘moderate’ risk of bias, primarily due to limitations in blinding, confounding, and reporting. Assessment of intervention fidelity across the included studies was limited. Based on the TIDieR checklist, several key items were reported inconsistently. Detailed information regarding provider training, adherence strategies, tailoring, or modifications over time was often incomplete or missing. Only a minority of studies reported on fidelity-enhancing strategies such as supervision, standardized protocols, or monitoring of intervention adherence. This lack of transparency impairs the replicability of interventions and limits the ability to assess whether observed effects were attributable to the intended intervention or to deviations from the planned delivery. Furthermore, substantial heterogeneity in study populations, timing and delivery of interventions, and outcome measures limits comparability and complicates synthesis. These limitations restrict the certainty and applicability of the current evidence and highlight the need for adequately powered, methodologically robust trials in diverse healthcare settings. Given the clinical heterogeneity inherent to post-sepsis syndrome, it remains to be elucidated whether the evaluated interventions yield consistent benefits across varying patient subgroups and care settings. Finally, no study specifically targeting the treatment of cognitive impairments could be identified, despite evidence suggesting that approximately two-thirds of sepsis survivors are affected by such deficits [5].

### Potential biases in the review process

Despite adhering to PRISMA guidelines and using a prospectively registered protocol, several potential sources of bias in the review process should be acknowledged. First, the restriction to publications in English and German may introduce language bias, potentially excluding relevant studies published in other languages. Second, although a comprehensive search strategy was applied across three major databases and supplemented by reference screening, there is a residual risk of publication bias, particularly as unpublished studies and grey literature were not systematically included beyond ClinicalTrials.gov. Third, while independent dual screening and data extraction were conducted to minimize selection and extraction bias, the reliance on subjective judgment in interpreting eligibility criteria, especially in mixed patient populations, may still introduce reviewer bias. Another potential source of bias arises from an adaptation made during the review process regarding the eligibility of mixed-population studies. Initially, the protocol did not predefine an inclusion threshold for studies enrolling both sepsis and non-sepsis patients, as it was not anticipated that mixed populations would be so common in this field. To ensure sufficient coverage of the available evidence while maintaining relevance to the target population, we subsequently decided to include studies in which at least approximately 50% of participants were sepsis survivors. While this approach balances inclusiveness and specificity, it introduces a potential source of selection bias and heterogeneity, as outcomes may be influenced by the characteristics of the non-sepsis subgroups within these studies.

### Relation to previous research

Several systematic reviews have already investigated interventions aimed at improving long-term outcomes in patients affected by post-intensive care syndrome (PICS) and recovering from severe COVID-19 [32–35]. These reviews similarly demonstrated that early rehabilitation, physical therapy, and multidisciplinary care models can positively impact physical function, fatigue, and quality of life. With our study, we aim to extend this research by focusing specifically on sepsis survivors, a group that shares many post-ICU vulnerabilities but has received comparatively less targeted attention in interventional research. This population includes both ICU- and non-ICU-treated patients, reflecting the full clinical spectrum of sepsis.

### Study limitations

This review has several limitations. First, the included studies are heterogeneous in terms of study design, intervention type, timing, duration, and outcome measures, which limited the ability to perform a meta-analysis and precluded direct comparisons across studies. Although we calculated standardized effect size measures to enhance comparability, the comparability of effect estimates is limited by the non-collapsibility of adjusted hazard ratio and adjusted odds ratios from conditional logistic regression models and survival regression models with different sets of covariates [17]. Furthermore, the comparability of standardized effects measures for metric outcomes we calculated from the studies is also limited due to the different outcomes and standardization methods. Second, sepsis definitions used varied considerably between studies, and some studies only included ICU- or ED-treated sepsis patients, which may limit the generalizability of the results of our review. Third, some randomized trials were pilot or feasibility studies with small sample sizes and short follow-up periods, increasing the risk of small study effects.

## Conclusions

This systematic review indicates that post-sepsis interventions such as rehabilitation programs and care coordination may offer meaningful benefits for survivors. They can improve common adverse outcomes of sepsis survivorship including long-term mortality, hospital readmissions and impairments in physical function. Technology-delivered psychological interventions may be a promising and scalable approach in future post-sepsis care, with the potential to reduce disparities in follow-up support for sepsis survivors. However, the current evidence base is limited by methodological weaknesses, small sample sizes, and substantial heterogeneity in both interventions and outcome measures. Importantly, no studies specifically addressed interventions for cognitive impairments, despite their high prevalence among survivors of sepsis. (Table 4)

Future research should focus on rigorously designed, adequately powered trials that assess multimodal, interdisciplinary interventions tailored to the complex and diverse needs of sepsis survivors with longer-term follow-up. To ensure that studies capture the domains most relevant to patients and health care providers, established core outcome sets for survivors of critical illness and sepsis should guide outcome selection [36, 37]. For health claims data-based analyses, the introduction of an ICD-10-code for sepsis aftercare or the Post-Sepsis-Syndrome could be beneficial [38]. In parallel, healthcare systems should work towards the integration of structured, long-term follow-up pathways– starting at hospital discharge and continuing throughout recovery– that provide physical, psychological, and cognitive support for patients and actively involve caregivers, who play a crucial role in coordinating and supporting post-sepsis care.

**Table 1:** Description of included studies

Author/Title	Vlake et al. (The Netherlands, 2021) [27]
Study design	RCT (feasibility study)
Population	ICU patients of two ICUs in the Netherlands
Inclusion criteria	patients > = 18 years admitted for sepsis or septic shock, mechanically ventilated (≥ 24 h)
Exclusion criteria	inability to understand the Dutch language, GCS < 15 during inclusion, active delirium or psychologic trauma and stress or Cognitive Status (TICS) score below or equal to 27 during inclusion, epilepsy, severe psychiatric diseases, or deafness or blindness
Sepsis definition	hypotension (MAP < 65 mm Hg) requiring the administration of a vasopressor (NA) or a blood lactate level at or above 3.0 mEq/L in whom infection was confirmed during ICU stay
Number of pat.	42
Post-ICU follow-up interventions	ICU-specific virtual reality session (experience of different facets of the ICU stay and ICU treatment)
Control group	exposure to a nature virtual reality environment
Primary outcome	Feasibility and safety of ICU-VR

Author/Title	Gawlytta et al. (Germany, 2022) [20]
Study design	RCT
Population	patients seeking advice at the German Sepsis Aid, patients of the Mid-German Sepsis Cohort, advertisement in health journals and hospitals/rehabilitation clinics
Inclusion criteria	≥ 18 years, treated for sepsis on an ICU for > 5 days and discharged from ICU > 1 month ago, having a spouse (married or cohabited)
Exclusion criteria	no spouse, acute psychosis, suicidal ideation, neuroleptics, not being fluent in German or ongoing psychotherapeutic treatment elsewhere of at least one dyad member
Sepsis definition	self-reported sepsis, sepsis defined as organ dysfunction due to a dysregulated host response to infection
Number of pat.	34
Post-ICU follow-up interventions	internet-based, therapist-led partner assisted cognitive-behavioral writing therapy − 2 × 50 min internet-based writing assignments per week over 5-week period, support letter written by the spouse
Control group	waitlist group
Primary outcome	Change in PTSD symptom severity (PCL-5)

Author/Title	Batterham et al. (UK, 2014) [19]
Study design	MCT
Population	ICU patients of two ICUs in UK
Inclusion criteria	18–65 year, a minimum of 3 days of ventilator support for trauma or sepsis, discharged home within 6 months of hospital admission
Exclusion criteria	inability to climb a flight of stairs, enrolment in another rehabilitation programme, and medical contraindication to cardiopulmonary exercise testing
Sepsis definition	no explicit sepsis definition mentioned
Number of pat.	59 (31 sepsis patients)
Post-ICU follow-up interventions	two hospital-based, physiotherapist-led supervised sessions per week. During the supervised sessions, participants exercised individually or in pairs for 40 min on a cycle ergometer (moderate exercise level)
Control group	usual care
Primary outcome	anaerobic threshold, physical function and mental health (SF-36 questionnaire v.2)

Author/Title	Winkler et al. (Germany, 2023) [25]
Study design	RCS with IPTW
Population	beneficiaries of a nationwide health insurance provider covering 30% of the German population
Inclusion criteria	> 15 years, sepsis, 6-months-survivors, unemployed at admission
Exclusion criteria	sepsis in the 2 years before their first hospitalization with sepsis in 2013–2014, employed at the time of hospital admission, pre-sepsis malignancy, severe nursing dependency, palliative care, dementia, pregnancy
Sepsis definition	ICD-10 German modification discharge codes (explicit sepsis codes)
Number of pat.	12,690
Post-ICU follow-up interventions	inpatient rehabilitation treatment
Control group	no rehabilitation treatment
Primary outcome	survival 7–12 and 13–36 months after discharge

Author/Title	Rahmel et al. (Germany, 2020) [15]
Study design	RCS matched
Population	benefiacries of a German health insurance provicer
Inclusion criteria	sepsis and septic shock
Exclusion criteria	pre-existing end-stage renal disease, home respiratory care, severe dementia, or a delirium 12 month before the index
Sepsis definition	ICD-10 German modification discharge codes
Number of pat.	167,948
Post-ICU follow-up interventions	inpatient rehabilitation treatment
Control group	discharge to home and self-care
Primary outcome	5-year post-discharge mortality

Author/Title	Chao et al. (Taiwan, 2014) [16]
Study design	RSC propensity score-matched
Population	Nationwide health insurance database covering 99% of the Taiwanese population
Inclusion criteria	patients admitted into the hospital between January 2000 and December 2011, diagnosis sepsis, survived for 90 days after discharge
Exclusion criteria	age < 18 years, no ICU admission, in-hospital death, death within 90 days after discharge
Sepsis definition	ICD-9-CM code 038.x (septicemia) and prescription of antibiotics
Number of pat.	31,070
Post-ICU follow-up interventions	rehabilitation within 90 days after discharge
Control group	propensity score-matched subjects who did not receive rehabilitation within 3 months after discharge
Primary outcome	mortality during 10-year follow-up

Author/Title	Schmidt et al. (Germany, 2016) [21]
Study design	RCT
Population	patients of 9 ICUs in Germany
Inclusion criteria	adult (≥ 18 years) survivors of severe sepsis or septic shock and fluent in the German language
Exclusion criteria	cognitive impairment
Sepsis definition	ACCP/SCCM consensus criteria (severe sepsis)
Number of pat.	291
Post-ICU follow-up interventions	case management with proactive patient symptom monitoring, clinical decision support for the PCP, and training for both patients and their PCPs in evidence-based care
Control group	usual care provided by primary care physician
Primary outcome	Change in mental HRQoL (SF-36 MCS) at 6 months

Author/Title	Schmidt et al. (Germany, 2020) [22]
Study design	RCT, follow-up
Population	patients of 9 ICUs in Germany
Inclusion criteria	adult (≥ 18 years) survivors of severe sepsis or septic shock and fluent in the German language
Exclusion criteria	cognitive impairment
Sepsis definition	ACCP/SCCM consensus criteria (severe sepsis)
Number of pat.	186
Post-ICU follow-up interventions	case management focusing on proactive patient symptom monitoring, clinical decision support for the primary care physicians (PCP) by a consulting physician, and training for both patients and their PCPs in evidence-based post-sepsis care
Control group	usual care provided by primary care physician
Primary outcome	Change in mental health-related quality of life (SF-36 MCS) at 6 months

Author/Title	Taylor et al. (USA, 2022) [28]
Study design	RCT
Population	adults treated with antibiotics in high risk for either 30-day readmission or mortality
Inclusion criteria	(1) age 18 years old or older; (2) antibiotic or bacterial culture order within 24 h of ED arrival and either (a) culture drawn first and antibiotics ordered within 48 h or (b) antibiotics ordered first and culture ordered within 48 h; (3) remaining hospitalized at the time a daily list of eligible patients was generated each weekday morning; and (4) deemed high risk for either 30-day readmission or mortality, defined as a readmission risk probability greater than or equal to 20% or mortality risk probability greater than or equal to 10%
Exclusion criteria	transferred from other acute care hospitals; DNR/DNI-status within 24 h after admission; resided more than 2.5 hours’ drive time from the treating hospital; or previously randomized to either treatment arm
Sepsis definition	suspected sepsis defined as antibiotic therapy or bacterial culture order within 24 h of ED arrival
Number of pat.	691
Post-ICU follow-up interventions	nurse navigators to deliver best practice care for sepsis survivors via telehealth including (1) identification and treatment of new physical, mental, and cognitive deficits; (2) review and adjustment of medications; (3) surveillance of treatable conditions that commonly lead to poor outcomes, including chronic conditions that may destabilize during sepsis and recovery; and (4) focus on care alignment, including palliative care when appropriate (STAR program)
Control group	usual transitional and outpatient care
Primary outcome	30 day-composite mortality or hospital readmission

Author/Title	Kowalkowski et al. (USA, 2022) [31]
Study design	RCT, follow-up
Population	patients of 3 US hospitals
Inclusion criteria	see Taylor et al. 2022
Exclusion criteria	see Taylor et al. 2022
Sepsis definition	see Taylor et al. 2022
Number of pat.	691
Post-ICU follow-up interventions	STAR program
Control group	usual transitional and outpatient care
Primary outcome	12-month all-cause hospital readmission

Author/Title	Colucciello et al. (USA, 2023) [29]
Study design	RCT, secondary analysis
Population	patients of 3 US hospitals
Inclusion criteria	see Taylor et al. 2022, aditionally: sepsis survivors discharged to post-acute care facilities
Exclusion criteria	see Taylor et al. 2022
Sepsis definition	suspected sepsis defined as antibiotic therapy or bacterial culture order within 24 h of ED arrival with high risk for mortality or readmission, included also patients with sepsis without organ dysfunction
Number of pat.	175
Post-ICU follow-up interventions	STAR program
Control group	usual transitional and outpatient care
Primary outcome	30-day composite outcome of all-cause mortality or hospital readmission

Author/Title	Deb et al. (USA, 2019) [14]
Study design	RCS
Population	Medicare beneficiaries
Inclusion criteria	short-term general and critical access hospital stays with an explicit or implicit sepsis diagnosis, enrolled in Medicare Parts A and B, but not managed care, for at least 2 months after discharge or until death, and where home health care occurred as the first postacute service in the week after discharge
Exclusion criteria	end-stage renal disease, metastatic cancer, or no home health nursing visits in the week after discharge
Sepsis definition	ICD-9 sepsis diagnosis codes: 995.91 (sepsis without organ dysfunction); 995.92 (severe sepsis); and 785.52 (septic shock). Implicit cases included ≥ 1 ICD-9 bacterial or fungal infection codes and an organ dysfunction code.
Number of pat.	170,571
Post-ICU follow-up interventions	(1) a nursing visit within 2 days of hospital discharge and at least 1 more visit in the first posthospital week, but no early physician visit (nursing protocol); (2) at least 1 physician visit within a week of discharge but not the nursing protocol [medical doctor (MD) protocol]; (3) both nursing and MD protocols
Control group	no event
Primary outcome	30-day all-cause hospital readmission

Author/Title	Taylor et al. (USA, 2020) [26]
Study design	RCS
Population	patients of 10 US hospitals
Inclusion criteria	adults with diagnosis of sepsis
Exclusion criteria	death during hospitalization, discharge into hospice
Sepsis definition	ICD-10 codes A40-41 and R65.2
Number of pat.	189
Post-ICU follow-up interventions	postsepsis care elements including medication optimization, screening for impairments, prevention and monitoring preventable causes of deterioration, treatment alignment with patient preferences
Control group	none or 1 postsepsis care element
Primary outcome	90-day all-cause mortality or unplanned hospital readmission

Author/Title	Mohammad et al. (USA, 2022) [18]
Study design	RCS
Population	1 ICU recovery clinic in the US
Inclusion criteria	adult ICU survivors with sepsis/septic shock and/or respiratory failure
Exclusion criteria	age < 18 years
Sepsis definition	no explicit sepsis definition mentioned
Number of pat.	104 (51 sepsis patients)
Post-ICU follow-up interventions	presence of pharmacist in an ICU-recovery clinic team
Control group	historical matched control group
Primary outcome	number of MRPs within 6 months of post-discharge

**Table 2:**
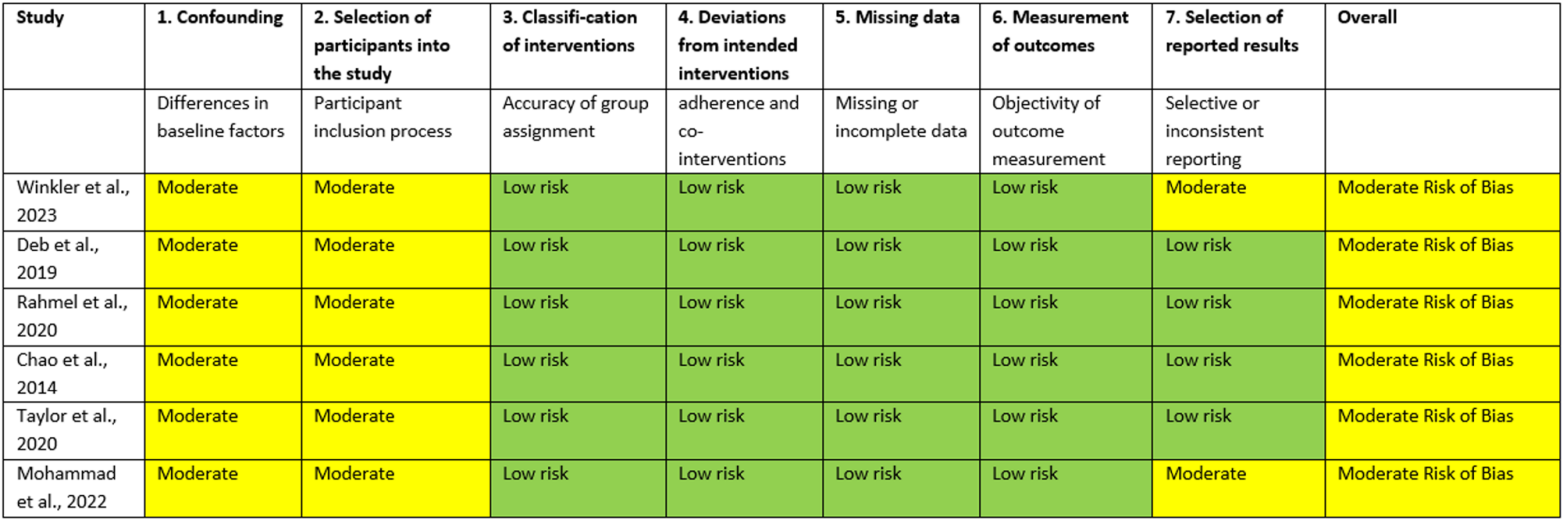
Risk of bias estimated using ROBINS-I-Tool

**Table 3:**
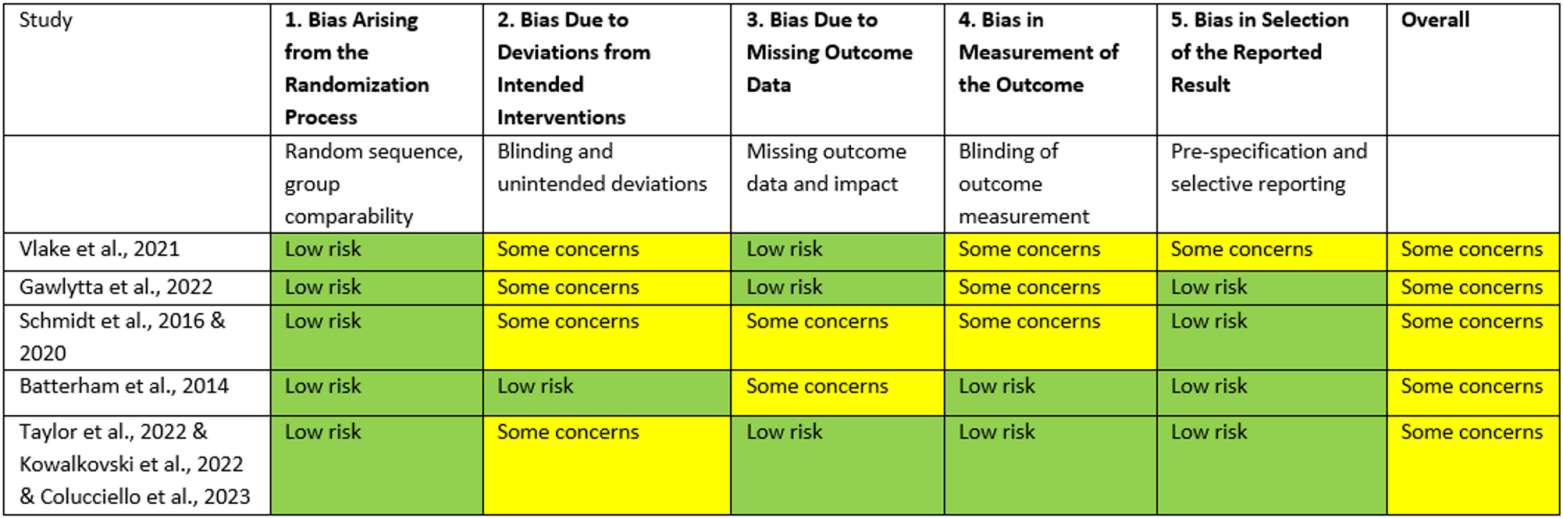
Risk of bias estimated using ROB2-Tool

**Table 4:** Summary of findings

Rehabilitation interventions to improve survival, physical function and mental health post-sepsis						
patients: all sepsis or ICU-treated sepsis patients setting: inpatient rehabilitation clinics or hospitals intervention: inpatient rehabilitation comparison: usual care						

Outcomes		Comparative risks/means		Relative effect	Number of participants	Comment
		*Control Group*	*Intervention Group*			
**Mortality or survival**						
7-12-months survival (Winkler)	OC	90.9	93.5	aOR 1.5 (95% CI, 1.2 to 1.7)^a^	12,690	
1-year mortality (Chao)	OC	34.0	31.3	aHR 0.92 (95% CI, 0.88 to 0.96)^b^	31,070	Unadjusted risks
5-year mortality (Rahmel)	OC	53.4	59.6	aHR 0.81 (95%-CI, 0.77 to 0.85)^c^	167,948	Unadjusted risks
5-year mortality (Chao)	OC	23.4	21.9	aHR 0.94 (95% CI, 0.91 to 0.97) ^b^	31,070	Unadjusted risks
10-year mortality (Chao)	OC	20.6	22.0	aHR 0.94 (95% CI, 0.92 to 0.97) ^b^	31,070	Unadjusted risks
**Nursing care dependency**						
7–12 months nursing care depencency (Winkler)	OC	48.2	47.0	aOR 1.0 (95% CI, 0.9 to 1.0)^a^	12,690	
**Anaerobic threshold**						
9-week AT (Batterham)	MCT	Mean: 10.7	Mean: 12.5	SMD = 0.58 (95% CI, 0.13 to 1.03)^d^	59	Subgroup of ICU-treated patients
**Physical function**						
9-week physical function SF-36 v2 PF (Batterham)	MCT	Mean: 40.1	Mean: 43.5	SMD = 0.26 (95% CI, −0.11 to 0.62)^d^	59	Subgroup of ICU-treated patients
**Mental health**						
9-week mental health SF36 MH (Batterham)	MCT	Mean: 47.9	Mean: 49.8	SMD = 0.15 (95% CI, −0.32 to 0.63)^d^	59	Subgroup of ICU-treated patients

Mental health interventions						
patients: ICU-treated sepsis patients setting: home or clinic intervention: digital-health intervention (VR, online writing therapy) comparison: waiting list or control VR						

Outcomes		Comparative risks/means		Relative effect	Number of participants	Comment
		*Control group*	*Intervention group*			
**PTSD**						
6-months IES-R (Vlake)	RCT	Mean: 23	Mean: 7	reported quantities are not sufficient to derive any standardized effect size measures	42	Subgroup of ICU-treated patients
6-months PCL-5 (Gawlytta)	RCT	Median: 20	Median: 22	SMD(CS) = −0.14 (95% CI, −0.81 to 0.54)	34	Subgroup of ICU-treated patients
Depression						
6-months BDI (Vlake)	RCT	Mean: 13	Mean: 6	Reported quantities are not sufficient to derive any standardized effect size measures	42	Subgroup of ICU-treated patients
6-months BSI-18 (Gawlytta)	RCT	Median: 12	Median: 13	SMD(CS) = 0.04 (95% CI, − 0.64 to 0.71)	34	Subgroup of ICU-treated patients

Care coordination						
patients: all or ICU-treated sepsis patients setting: outpatient or clinics intervention: nurse- or GP-led interventions, bundle strategies comparison: standard care						

Outcomes		Comparative risks/means		Relative effect	Number of participants	Comment
		*Control group*	*Intervention group*			
**Readmission or mortality**						
30-days readmission (Deb)	OC	Not reported	Not reported	adjusted risk reduction: 7% (95% CI, 2 to 12%)	170,571	Both protocols vs. none
30-days readmission or mortality (Taylor 2022)	RCT	30.3	25.5	aOR 0.80 (95% Cl, 0.64 to 0.98)^e^	691	
30-days readmission or mortality (Colucciello)	RCT	30.6	20.7	aOR 0.57 (0.28 to 1.16)^e^	175	Subgroup of patients discharged to post-acute care
90-days readmission or mortality (Taylor 2020)	OC	Not reported	Not reported	aOR 0.26 (95% CI, 0.10 to 0.69)^f^	189	Two elements vs. none
90-days readmission or mortality (Taylor 2020)	OC	Not reported	Not reported	aOR 0.28 (95% CI, 0.11 to 0.72)^f^	189	Three elements vs. none
90-days readmission or mortality (Taylor 2020)	OC	Not reported	Not reported	aOR 0.12 (95% CI, 0.03 to 0.50)^f^	189	Four elements vs. none
1-year readmissions or mortality (Kowalkowski)	RCT	65.0	72.2	aOR 0.70 (95% CI, 0.50 to 0.98)^e^	691	
**Mental health**						
6-months SF-36 MCS Score (Schmidt 2016)	RCT	MCS(control) = 1.64 (95% CI, −1.70 to 6.09) SMCS(control) = 0.13 (95% CI, −0.14 to 0.48)	MCS(intervention) = 3.79 (95% CI, 1.05 to 6.54) SMCS(intervention) = 0.30 (95% CI, 0.08 to 0.52)	SMD(CS) = 0.17 (95% CI, −0.14 to 0.49)	291	Subgroup of ICU-treated sepsis
24-months SF-36 MCS Score (Schmidt 2020)	RCT	MCS: 1.1 [13.6]	MCS: 3.1 [13.9]	SMD(CS) = 0.15 (95% CI −0.16 to 0.45)		Difference to baseline, subgroup of ICU-treated sepsis
6-months PTSS-10 (Schmidt 2016)	RCT	MCS − 0.2	MCS: −2.0 (11.0)	SMD(CS) = −0.16 (95% CI, −0.43 to 0.11)	291	Subgroup of ICU-treated sepsis
24-months PTSS-10 (Schmidt 2020)	RCT	MCS: −0.7	MCS: 3.7	SMD(CS) = −0.37 (95% CI, −0.66 to −0.01)		Difference to baseline, subgroup of ICU-treated sepsis
**Physical function**						
6-months XSFMA-F (Schmidt 2016)	RCT	Mean: 46.9	Mean: 38.0	SMD=−0.25 (95% CI, −0.48 to −0.02)	291	Subgroup of ICU-treated sepsis
6-months XSFMA-B (Schmidt 2016)	RCT	Mean: 52.4	Mean: 42.5	SMD= −0.27 (95% CI, −0.50 to −0.04)	291	Subgroup of ICU-treated sepsis
6 months ADL (Schmidt 2016)	RCT	Mean: 8.6	Mean: 7.6	SMD = 0.27 (95% CI, 0.04 to 0.50)	291	Subgroup of ICU-treated sepsis
24-months XSFMA-F (Schmidt 2020)	RCT	Median: 25.0	Median: 20.8	SMD= −0.11 (95% CI, −0.49 to 0.27)		Subgroup of ICU-treated patients
24-months XSFMA-B (Schmidt 2020)	RCT	Median: 25.0	Median: 18.75	SMD= −0.07(95% CI, −0.29 to 0.15)		Subgroup of ICU-treated patients
24-months ADL (Schmidt 2020)	RCT	Median: 8.8	Median: 8.9	SMD = 0.04 (95% CI, −0.27 to 0.35)		Subgroup of ICU-treated patients

Pharmacist-led interventions						
patients: ICU-treated sepsis patients setting: ICU-recovery clinic intervention: pharmacist in the ICU-RC team comparison: pre-intervention						

Outcomes		Comparative risks/means		Relative effect	Number of participants	Comment
		*Control group*	*Intervention group*			
**Medication related problems**						
6-months number of medication-related problems (Mohammad)	OC	Mean: 1.6	Mean: 2.1	SMD = 0.04 (95% CI, −0.27 to 0.35)	104	Unadjusted

a - age; sex; pre-existing comorbidities according to Charlson and Elixhauser Comorbidity Scores; prior asplenia, immobility, mechanical ventilation, and dialysis; prior health care utilization and costs in the 12 months pre-sepsis; and characteristics of the acute septic disease (focus of infection, type of organ dysfunction), and therapies during index hospitalization;

b - age, sex, year and month of index date, monthly income, urbanization, CCI, hypertension, dyslipidemia, heart failure, myocardial infarction, cerebrovascular disease, diabetes mellitus, plegia, dementia, and sepsis severity;

c - age, sex, CCI, year of admission, nursing dependency at admission, number of comorbidities, hospital length of stay;

d - baseline outcome (pre-test), age, sex, and cause of entry to ICU, study site;

e - age, comorbidity burden, organ dysfunction at enrolment;

f - age, comorbidity, length of stay, discharge disposition;

otherwise not specified

Abbreviations: a– adjusted, HR– hazard ratio, OR– odds ratio, AT - anaerobic threshold; SF-36 PF - Short Form 36 version 2, Physical function; SF-36 MH - Short Form 36 version 2, Mental health; IES-R - Impact of Event Scale-Revised; PCL-5 - PTSD-Checklist for DSM-5, SMD– standardized mean difference, CS– change score, BDI - Beck Depression Inventory, BSI 18 - Brief Symptom Inventory, SF-36 MCS– SF-36 Mental Component Summary, XSFMA-F/B Extra Short Musculoskeletal Function Assessment for physical function (F) and disability (B); ADL - Activities of Daily Living, PTSS-10 - Post-traumatic Symptom Scale, OC– observational study, RCT– randomized controlled trial, MCT– minimalized controlled trial, MCS - mean change score (pre-post); SMCS - standardized mean change score = MCS/SD(baseline) = Change in SD between T0 (baseline) and T1 (post); SMD(CS) - standardized mean differences in CHANGE-SCORES

## Supplementary Information

Supplementary Material 1

## Data Availability

No datasets were generated or analysed during the current study.

## Abbreviations

ACCP: American college of chest physicians
ADL: Activities of daily living
CI: Confidence intervals
ED: Emergency department
HR: Hazard ratio
ICD: International statistical classification of diseases and related health problems
ICU: Intensive care unit
iCBT: Cognitive behavioral writing therapy
IES-R: Impact of events scale–revised
IQR: Interquartile ranges
MD: Mean difference
OR: Odds ratio
PICS: Post-intensive care syndrome
PSS: Post-sepsis syndrome
PRISMA: Preferred reporting items for systematic reviews and meta-analyses
PTSD: Post-traumatic stress disorder
RCT: Randomized controlled trials
RoB: Risk of bias
SD: Standard deviation
SMD: Standardized mean differences
SMOOTH: Sepsis survivors monitoring and coordination in outpatient health care
SSCM: Society of critical care medicine
STAR: Sepsis transition and recovery
US: United States of America
VR: Virtual reality
XSMFA-B: Extra Short Musculoskeletal Function Assessment regarding disability
XSMFA-F: Extra Short Musculoskeletal Function Assessment regarding physical function
