# Supplementary material for: Effectiveness of targeted post-acute interventions and follow-up services for sepsis survivors: a systematic review
Source: Crit Care. 2025 Aug 8;29:351. doi: 10.1186/s13054-025-05585-3 (PMC12333265; doi:10.1186/s13054-025-05585-3)
Supplement: Supplementary file 1 — Supplementary Material 1 [file 13054_2025_5585_MOESM1_ESM.docx]

**Supplement**

**Search strategy:**

1. Pubmed:

(("sepsis"[Title/Abstract] OR "septic shock"[Title/Abstract]) AND ("survivors"[All Fields] OR "post-sepsis syndrome"[All Fields] OR "PICS"[All Fields] OR "postintensive care syndrome"[Supplementary Concept] OR "postintensive care syndrome"[All Fields] OR "post intensive care syndrome"[All Fields]) AND ("intervention*"[All Fields] "discharge"[All Fields] OR "follow-up"[All Fields] OR "rehabilitation"[All Fields] OR "clinic*"[All Fields] OR "bundle*"[All Fields] OR ("aftercare"[MeSH Terms] OR "aftercare"[All Fields]) OR "long-term"[All Fields] OR ("guidance"[All Fields] OR "guidances"[All Fields]) OR "coordinat*"[All Fields])) AND (2013:2024[pdat])

1. Web of science:

(((AB=(sepsis OR septic shock)) AND ALL=("survivors" OR "post-sepsis syndrome" OR "PICS" OR "postintensive care syndrome" OR "post intensive care syndrome")) AND ALL=("intervention*" OR "discharge" OR "follow-up" OR "clinic*" OR "bundle*" OR "aftercare" OR "rehabilitation" OR "long-term" OR "guidance" OR "guidances" OR "coordinat*")) AND PY=(2013-2024)

1. Clinicaltrials.gov:

("Brain Dysfunction" OR "Cognitive Impairment" OR "Septic Shock") AND ("Behavioral: Physical and functional rehabilitation" OR "Cognitive rehabilitation" OR "Transcutaneous electrical stimulation") AND "Interventional" AND "Completed"

eTable 1: Description of study protocols

| Reference | O'Connor et al. (USA), 2022 |
| --- | --- |
| Design | RCT |
| Duration of intervention | 12 months |
| Number of participants | 400 |
| Inclusion criteria | sepsis survivors |
| Exclusion criteria | none mentioned |
| Description of intervention | home health care (nursing visits), outpatient physician care (1+ evaluation/care within 7 days) |
| Reference | Schmidt et al. (Germany), 2014 |
| Design | RCT |
| Duration of intervention | 12 months |
| Number of participants | 290 |
| Inclusion criteria | survivors of severe sepsis or septic shock |
| Exclusion criteria | insufficient German language, deafness, blindness, speech impairment, severe cognitive impairment |
| Description of intervention | discharge management with structured information, training of GPs and patients, monthly telephone monitoring |
| Reference | Kowalkowski et al. (USA), 2021 |
| Design | RCT |
| Duration of intervention | during hospitalization |
| Number of participants | 4032 |
| Inclusion criteria | ≥18 y upon admission; clinically suspected infection; organ dysfunction, defined as two or more points on SOFA or quick-SOFA; high risk of hospital readmission within 90 days; not discharged at the time the daily list of eligible patients is generated |
| Exclusion criteria | change in code status (DNR) within 24h after presentation, reside > 2.5h drive from treating hospital, active participants in different AH care management program, previously randomized in this study |
| Description of intervention | proactive coordination and monitoring to patients using targeted, evidence-based best-practice care, telephone- and EHR-based support within the hospitalization and to patients across all discharge settings with remote follow-up at specified intervals |
| Reference | Wójcik et al. (Poland), 2019 |
| Design | Patient-centered trial based on personalized medicine |
| Duration of intervention | 3 months |
| Number of participants | 60 |
| Inclusion criteria | patients 30–80 years of age with a documented episode of sepsis |
| Exclusion criteria | sepsis and trauma, cancer or burn injury background, with leg amputations, requiring palliative care, psychiatric or with mental disability |
| Description of intervention | 3-month diagnostically monitored rehabilitation programs based either on the recumbent cycloergometer training or on the experimental hyperbaric oxygen therapy |
| Reference | Kowalkowski et al. (USA), 2019 |
| Design | Study Protocol RCT |
| Duration of intervention | 30 days after discharge |
| Number of participants | 708 |
| Inclusion criteria | ≥18 y; antibiotic or bacterial culture order within 24 hours of ED arrival and either (a) culture drawn first and antibiotics ordered within 48 hours or (b) antibiotics ordered first and culture ordered within 48 hours; remaining hospitalized at the time a daily list of eligible patients was generated; and high risk for either 30-day readmission or mortality |
| Exclusion criteria | transferred from other acute care hospitals; DNR/DNI-status within 24 hours after admission; resided more than 2.5 hours’ drive time from the treating hospital; or previously randomized to either treatment arm, ruled out infection |
| Description of intervention | nurse navigators to deliver best practice care for sepsis survivors via telehealth including (1) identification and treatment of new physical, mental, and cognitive deficits; (2) review and adjustment of medications; (3) surveillance of treatable conditions that commonly lead to poor outcomes, including chronic conditions that may destabilize during sepsis and recovery; and (4) focus on care alignment, including palliative care when appropriate; 15 times over the study period for a total of 170 minutes |

eTable 2: TIDierR Checklist for complex interventionen

| **TIDieR Item** | **SMOOTH (Schmidt 2016/19)** | **ICU-VR (Vlake)** | **STAR (Taylor 2022)** | **REPAIR (Gawlytta)** | **PIX (Batterham)** |
| --- | --- | --- | --- | --- | --- |
| 1. Name | Primary-care management intervention | ICU-specific VR | Sepsis Transition and Recovery (STAR) program | Internet-based therapist-led cognitive-behavioural writing therapy (iCBT) | Hospital-based supervised aerobic exercise rehabilitation |
| 2. Why (Rationale) | Improve mental-health–related QoL | Reduce PTSD and improve understanding of ICU (feasibility study: feasibility and safety) | Reduce 30-day mortality and readmissions post-sepsis | Reduce PTSD in sepsis survivors & spouses | Aimed to improve physical fitness and QoL in critical illness survivors |
| 3. Materials | Training materials for patients & PCPs; case-management protocols; phone monitoring | VR headset + content software | Phone protocols, checklists | Secure web portal for delivery; redefined writing modules, therapist’s written feedback | Hospital cycle ergometers, training protocols for supervised sessions and guidance for an unsupervised weekly session |
| 4. Procedures | 12-month intervention: PCP + patient training, nurse case management, clinical support for PCPs, periodic phone check-ins | multiple VR sessions post-ICU (at least one, median 1 session) | 30-day multicomponent activities: care coordination, symptom management, medication management, chronic disease follow-up, palliative care, social support | 10 online writing sessions in 5 weeks, spouse support letter | 8-week intervention, 2 supervised 30-minute sessions/week + 1 unsupervised session of equivalent duration |
| 5. Providers | Trained nurses (case managers); liaison physician trained in sepsis aftercare; trained PCPs | supervised sessions; provider not specified | Specialized nurse navigators, Supervision by a medical director; integration with hospital transition service and PCPs | Trained therapists (not further unspecified) via web | Physiotherapists: supervised sessions in hospitals |
| 6. Modality | In-person GP + telephone + clinical support by liaison physicians | In-person VR | primary mode is virtual, the infrastructure supports potential in-person visits if clinically indicated | Asynchronous online | Face-to-face, individually or in pairs; Components of self-directed home sessions described verbally. |
| 7. Setting | Multicenter German primary-care practices, home | hospital ward (post-ICU) | Multicenter hospitals in the USA | Remote (Germany) via internet | Hospital-based ICU follow-up clinics, home |
| 8. Dose/Schedule | 12 months of coordinated care + phone calls at regular intervals (monthly contact in the first six months, contact every three months thereafter) | Session frequency varies based on patient preferences, ≥1 session per patient | Daily/weekly for 30 days, initial Contact: Immediately post-discharge, follow-Up Contacts: Regularly scheduled calls/visits | 2 × 50 min/week for 5 weeks | 8 weeks total, scheduled at 2 supervised sessions/week + 1 home session, each lasting ~30 minutes. |
| 9. Tailoring | Tailored to patient risk via PCP assessment and case manager symptom monitoring | VR-content and session number chosen by participants; no formal tailoring | Dose tailored based on the risk profile and needs of each patient, detailed process evaluation identified variability in how care packages were delivered (latent classes), highlighting real-world customization | Feedback-based tailoring; no formal protocol | Exercise intensity increase based on individual endurance improvement |
| 10. Fidelity | 70% delivery with high fidelity in intervention group; fidelity measured through adherence and health care utilization metrics, reasons for non-fidelity: death of patients | all included patient completed at least one VR-session with varying frequency | Navigators engaged patients a median of 15 times over the study period for a total of 170 minutes, the proportion of patients with goal-concordant care was higher in the intervention group for all elements of the STAR intervention | Dropout tracked (~20%), dropouts due to physical deterioration, change in life circumstances, patient interviews confirmed the applicability of the intervention, no technical issues | Mean supervised sessions attended 12/16; mean unsupervised sessions 6/8; approx. 50% completed all sessions; no formal fidelity assessment |
